# From dysphoria to anhedonia: age-related shift in the link between cognitive and affective symptoms

**DOI:** 10.1093/geronb/gbaf252

**Published:** 2025-12-10

**Authors:** Daniel Harlev, Aya Vituri, Moni Shahar, Noham Wolpe

**Affiliations:** Department of Physical Therapy, The Stanley Steer School of Health Professions, Gray Faculty of Medical & Health Sciences, Tel Aviv University, Tel Aviv, Israel; Department of Psychiatry, Rambam Health Care Campus, Haifa, Israel; Tel Aviv Center for Artificial Intelligence & Data Science (TAD), Tel Aviv University, Tel Aviv, Israel; Tel Aviv Center for Artificial Intelligence & Data Science (TAD), Tel Aviv University, Tel Aviv, Israel; Department of Physical Therapy, The Stanley Steer School of Health Professions, Gray Faculty of Medical & Health Sciences, Tel Aviv University, Tel Aviv, Israel; Sagol School of Neuroscience, Tel Aviv University, Tel Aviv, Israel

**Keywords:** Depression, Bridging, Cognition, Gray matter volume, Symptoms Network Analysis

## Abstract

**Objective:**

Depression in aging shows heterogeneous symptoms across cognitive, affective, and neurobiological domains. Traditional categorical diagnoses may not capture these complex patterns, prompting a shift toward dimensional or domain-based approaches. We examined whether the symptoms that bridge cognition and affect differ by age and explored their associations with brain structure.

**Methods:**

Data from 756 young (≤45 years) and 1,230 older (≥65 years) adults from the Cambridge Centre for Ageing and Neuroscience were analyzed. Cognition was assessed using the Addenbrooke’s Cognitive Examination—Revised, and depressive and anxiety symptoms were assessed using the Hospital Anxiety and Depression Scale. Graphical LASSO was used to construct cognitive–affective networks, testing for age-related differences in strength and bridging centrality measures. Building on these findings, we further examined the association between bridging symptoms, cognition, and gray matter volume (GMV).

**Results:**

Symptom strength centrality was similar across age groups. However, significant age-related differences emerged in bridging symptoms. Specifically, the primary bridging symptom differed, with dysphoria in young adults and anhedonia in older adults. Follow-up analyses showed that cognition mediated the link between GMV and anhedonia, but not dysphoria, particularly in older adults.

**Discussion:**

Cognitive–affective bridging symptoms differ with age, with anhedonia replacing dysphoria as the key bridge in older adults. This shift was linked to age-related differences in the relationship between GMV, cognition, and depressive symptoms. These results highlight the need to target different symptoms to alleviate cognitive–affective manifestations across the lifespan.

Traditional psychiatric diagnosis relies on categorical classifications, assigning diverse symptoms to single diagnostic entities such as Major Depressive Disorder (Regier et al., 2013). However, evidence increasingly shows that depressive disorders are highly heterogeneous: patients with the same diagnosis may present with very different symptom profiles, severities, and functional impairments (Fried, 2017; Goldberg, 2011). Such heterogeneity challenges the validity of categorical diagnoses and raises questions about their utility for understanding mechanisms, predicting outcomes, or guiding personalized treatment (Cuthbert & Insel, 2013).

These diagnostic challenges become even more pronounced in the context of aging. Older adults experience significant changes across cognitive, affective, and neurobiological domains, and mental health diagnoses such as depression and anxiety are common in this population (Bergmann et al., 2025; Teachman, 2006; Wang & Blazer, 2015). Depressive and anxiety symptoms in older adults often overlap with cognitive decline, both clinically and biologically, making it difficult to disentangle psychiatric syndromes from neurodegenerative processes (Alexopoulos, 2019; Butters et al., 2008; Unützer, 2007). This overlap highlights the limitations of categorical thinking, as symptoms may span multiple domains, such as apathy or anhedonia, and carry different implications depending on the individual (Fahed & Steffens, 2021; Harlev et al., 2025a; Husain & Roiser, 2018). Approaches that move beyond categorical labels toward understanding how specific symptoms interrelate, such as network analysis, may offer clearer insights into the complex mental health landscape in older age (Harlev et al., 2025b).

Symptom network analyses offer a powerful approach for understanding how specific symptoms interact and cluster into meaningful patterns across domains (Borsboom & Cramer, 2013; Boschloo et al., 2015). However, most existing studies have focused either on younger adults or on older adults in isolation, without comparing networks across the lifespan (Beard et al., 2016; Harlev et al., 2025b; Park & Kim, 2020). Moreover, many have examined either cognitive or affective domains separately, rather than investigating how these domains interact. In older adults, previous studies have focused on affective symptoms while excluding cognitive domains (An et al., 2019; Belvederi Murri et al., 2020), and the few studies combining affective and cognitive domains had methodological limitations. For example, one study (Bai et al., 2023) used less sensitive cognitive measures, like the Mini-Mental State Examination, which lacks sensitivity to specific cognitive domains, such as executive function, and is particularly limited in detecting mild cognitive impairment, a condition highly relevant in aging populations and often overlapping with affective symptoms (Ciesielska et al., 2016). Another study (Zhou et al., 2023) focused on clinical populations, limiting broader applicability to the general population. Finally, few studies have integrated neurobiological measures, such as gray matter volume (GMV), which are essential for linking symptom networks to underlying brain structure and understanding potential mechanisms driving cognitive–affective interactions.

Here, we address these gaps by directly comparing cognitive–affective networks in the general population across the lifespan. We analyzed the relationship between affective symptoms and cognitive domains using data from the Cambridge Centre for Ageing and Neuroscience (Cam-CAN), which is a population-based cohort of overall healthy individuals across the lifespan. Cognitive performance was assessed with the Addenbrooke’s Cognitive Examination—Revised (ACE-R) to assess performance in key cognitive domains. Affective symptoms were assessed with the Hospital Anxiety and Depression Scale (HADS). We compared strength centrality to assess overall network structure and bridge centrality measures to assess the cognitive–affective links in both young and older adults. We further tested whether depression and anxiety symptoms differentially contribute to linking cognitive and affective symptoms across age groups. Lastly, we explored whether cognitive performance mediates the association between GMV and depressive symptoms (Bergmann et al., 2025) and whether this mediation is moderated by age.

## Methods

### Participants

We analyzed data from the Cam-CAN, a large-scale project focusing on aging (Shafto et al., 2014). Cam-CAN recruited a population-based sample of approximately 2,600 adults aged 18 years and over from the general population via Primary Care Trust’s lists within the Cambridge City area in the United Kingdom. Ethical approval for the study was granted by the Cambridgeshire 2 Research Ethics Committee (reference: 10/H0308/50), and written informed consent was obtained from all participants prior to their participation. The dataset includes detailed demographic, cognitive, and neuroimaging data.

Participants were categorized into two age groups: young (≤45 years) and older (≥65 years), to test for age-related differences in cognitive–affective networks. Cognitive performance was assessed using the ACE-R (Mioshi et al., 2006), which evaluates five domains: memory, fluency, language, visuospatial abilities, and orientation. Affective symptoms were measured using the HADS (Bjelland et al., 2002), which includes subscales for depressive (HD1–HD7) and anxiety (HA1–HA7) symptoms. After excluding participants with missing demographic or clinical data, the groups included 756 young adults and 1,230 older adults.

### Network analyses

Sparsity-based networks were constructed separately for each age group using the Graphical LASSO method, as done previously (Friedman et al., 2008). We included participants across the full range of HADS scores, including those with low or zero scores, to capture the entire spectrum of affective symptomatology and its relationship with cognitive performance, as done previously (Bai et al., 2023). The use of Graphical LASSO allowed us to include all participants by penalizing weak or noisy connections to ensure network construction was robust across the full range of affective and cognitive scores. Non-significant connections were removed based on the Extended Bayesian Information Criterion (Foygel & Drton, 2010) for model selection. Nodes represented cognitive and affective domains, with edges reflecting regression coefficients. Covariates, including sex and education (number of years in education), were included to account for potentially confounding effects. All non-categorical data were z-scored before entry into the analysis.

In line with our study goals and with previous research, we computed two key metrics in the networks (Bringmann et al., 2019; Jones et al., 2021). First, we sought to identify the importance of each node within the whole network by calculating strength centrality. Strength centrality quantifies the total connectivity of a node by summing the absolute weights of all edges connected to it. Higher strength centrality indicates that a node is more integrated within the network, i.e., it has stronger connections to multiple other nodes. This allowed us to test whether the overall network structure remained similar with age, with similarly important nodes.

Second, we sought to identify the nodes linking cognitive and affective symptoms by computing two independent and complementary centrality measures. Betweenness centrality quantifies how often a node appears on the shortest paths between pairs of other nodes in a network (where shortest path is the minimum number of steps/lowest-weight sum needed to travel between two nodes in a network). In other words, betweenness centrality reflects a node’s role as an intermediary. When a node mediates a large proportion of the shortest paths, it plays a critical role in connecting otherwise separate regions of the network, and is thus interpreted as a bridging measure (Zhang & Luo, 2017). To complement this approach, we also computed Bridge Expected Influence (BEI), which measures a node’s connections spanning different pre-defined communities, weighted by their influence on neighboring nodes (Jones et al., 2021). Unlike betweenness centrality, BEI is domain-sensitive and requires a priori labeling of the different symptom domains (cognitive and affective).

We first assessed the consistency of centrality ranks across age groups using Spearman (rank) correlation to determine whether the overall structure of centrality values remained similar between young and older adults. Next, we reported the nodes with the highest centrality value in each domain (depression, anxiety, and cognition) for each age group to identify possible age-related differences in the importance of network nodes. Finally, we tested for differences in centrality measures between depression and anxiety nodes in each group. This tests whether, within each group, there were any significant differences in centrality measures between depression and anxiety symptoms. To test this, permutation testing with 5,000 iterations was used within each age group to compare centrality measures between depression-related (HD1–HD7) and anxiety-related (HA1–HA7) nodes. The test was performed by randomly shuffling the depression and anxiety labels while maintaining the original network topology, generating a “null” distribution of expected differences by chance alone. Statistical significance was determined as the proportion of permutations in which the shuffled difference exceeded the observed value (with *p *< .05 considered statistically significant).

#### Mediation and moderated mediation analyses

Building on the network analyses, we conducted exploratory post hoc moderated mediation analyses. Specifically, we tested whether cognition mediates the association between GMV and depressive symptoms and whether this mediation pathway is moderated by age. This analysis included 733 participants who took part in Cam-CAN Stage 2 and Cam-CAN Frail, which followed similar neuroimaging protocols in individuals with lower scores in the ACE-R (Kocagoncu et al., 2022; Shafto et al., 2014). Structural MRI data were acquired using a 3 T Siemens TIM Trio scanner with T1-weighted MPRAGE sequences (TR = 2,250 ms, TE = 2.99 ms, TI = 900 ms, flip angle = 9°, isotropic voxel size = 1 mm³) (Taylor et al., 2017). Preprocessing followed standard protocols in SPM12, including segmentation into gray matter. To account for individual differences in head size, total intracranial volume was computed from the segmentation, and GMV was included in the model as the ratio GMV/total intracranial volume.

We specified structural equation models in R using the lavaan package (Chambers, 2008). In the first step, cognition, measured by the ACE-R total score, was modeled as a mediator of the association between GMV and depressive symptoms (HD7 representing anhedonia and HD3 representing dysphoria), while controlling for sex and education. Indirect effects were estimated using 5,000 bootstrapped samples to derive confidence intervals. In the second step, we tested for a moderated mediation by including an interaction term between GMV and age on the path from GMV to cognition. All continuous predictors, including GMV, age, education, and ACE-R scores, were z-scored before being entered into the analyses.

All statistical analyses were conducted in Python (v3.11.5) (Van Rossum & Drake, 2009).

## Results

### Participants and item severity

Participant demographics are summarized in Table 1 for both young and older adult groups. Sex distribution was similar across groups, but education level was significantly higher for younger compared to older adults. Depressive symptoms varied between the two groups. Older adults had significantly higher scores on items related to psychomotor slowing and reduced anticipatory anhedonia/enjoyment, while young adults scored higher on measures of dysphoria/cheerfulness and consummatory anhedonia/enjoyment. There were no significant differences between groups for apathy/interest in appearance or laughter. In contrast, anxiety symptoms were consistently higher among young adults across all items. Lastly, young adults had better cognitive performance compared to older adults across all cognitive domains. To further characterize symptom distributions, we examined their overall range and skew across the sample. Consistent with a population-based design, depressive and anxiety symptoms were low on average and showed a large number of zero values, supporting the use of sparsity-based network estimation methods (Supplementary Section 1, Supplementary Table 1; see online supplementary material).

**Table 1.** Summary of demographics and item-wise comparisons between young and older adults.

Variables	Young adults (mean ± *SD* or %)	Older adults (mean ± *SD* or %)	Test statistic	*p* ^a^
**Age**	33.49 ± 6.87	79.29 ± 7.41		
**Sex**	42.9%	43.5%	1.26	.557
**Education^b^**			348.68	<.001
** None**	1.7%	29.5%		
** GCSE**	4.1%	7.7%		
** A level**	8.1%	3.1%		
** University**	86.1%	59.7%		
**Anhedonia/enjoyment (HD1)**	0.46 ± 0.67	0.61 ± 0.78	−4.65	<.001
**Laughter (HD2)**	0.26 ± 0.50	0.27 ± 0.54	−0.25	.802
**Dysphoria/cheerful feeling (HD3)**	0.35 ± 0.54	0.27 ± 0.52	3.11	.002
**Psychomotor retardation/slowed down (HD4)**	0.70 ± 0.72	1.36 ± 0.93	−16.47	<.001
**Apathy/loss of interest in appearance (HD5)**	0.50 ± 0.74	0.47 ± 0.71	0.85	.434
**Anticipatory anhedonia/look forward to things (HD6)**	0.37 ± 0.65	0.53 ± 0.72	−4.91	<.001
**Anhedonia/enjoyment of book/media (HD7)**	0.28 ± 0.63	0.19 ± 0.52	3.52	<.001
**Feeling of tension (HA1)**	1.06 ± 0.65	0.78 ± 0.62	9.52	<.001
**Frightened feeling (HA2)**	0.69 ± 0.81	0.53 ± 0.74	4.38	<.001
**Worrying thoughts (HA3)**	0.98 ± 0.87	0.76 ± 0.80	5.86	<.001
**Relaxed feeling (HA4)**	0.84 ± 0.68	0.60 ± 0.63	7.93	<.001
**Butterflies in stomach (HA5)**	0.61 ± 0.64	0.43 ± 0.59	6.49	<.001
**Restless feeling (HA6)**	1.21 ± 0.90	0.92 ± 0.82	7.36	<.001
**Feeling of panic (HA7)**	0.54 ± 0.67	0.49 ± 0.65	1.68	.107
**Memory**	0.93 ± 0.10	0.82 ± 0.17	15.81	<.001
**Fluency**	0.89 ± 0.13	0.78 ± 0.19	15.04	<.001
**Language**	0.94 ± 0.10	0.92 ± 0.10	3.65	<.001
**Visuospatial**	0.98 ± 0.05	0.91 ± 0.14	13.52	<.001
**Orientation**	0.97 ± 0.06	0.93 ± 0.10	10.15	<.001

*Note*. Demographic, cognitive, and affective items for young and older adults are presented. Continuous variables, including age, and cognitive and affective measures, are reported as mean ± standard deviation and compared using independent *t*-tests. Categorical variables, such as sex distribution and education, are expressed as percentages and compared with chi-square tests. HD and HA, followed by the number, indicate the subscale items for depressive and anxiety symptoms, respectively, in the Hospital Anxiety and Depression Scale.

a All *p*-values were corrected for multiple comparisons using false discovery rate (FDR) correction.

b Education was categorized according to the English system: “None” (no education beyond age 16), “GCSE” (General Certificate of Secondary Education), “A Levels” (General Certificate of Education Advanced Level), and “University” (undergraduate or graduate degree).

### Network analyses

**Figure 1.**
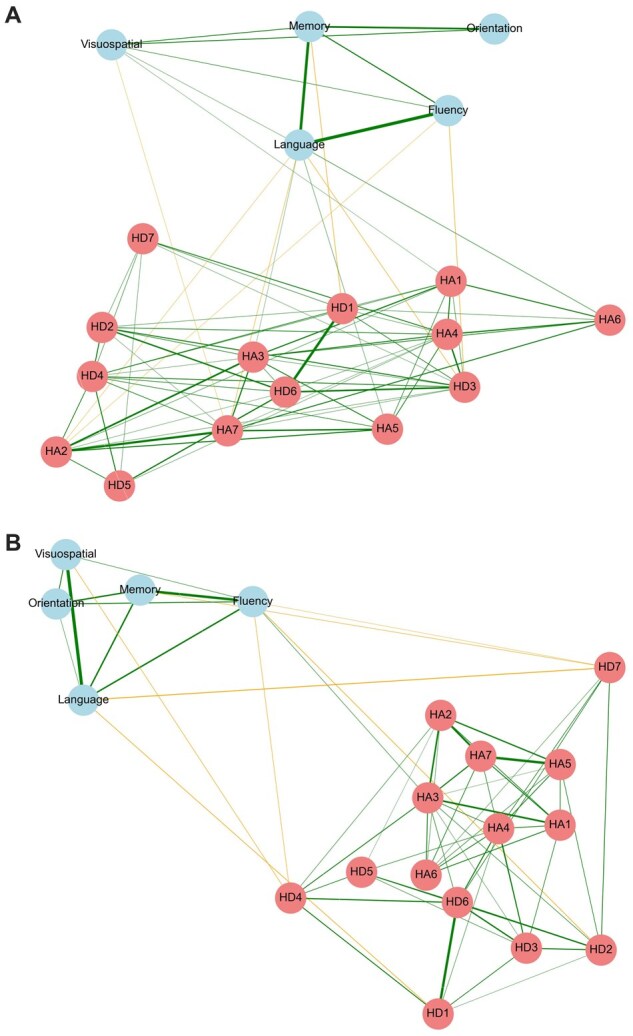
Sparsity-based networks for young and older adults. (A) Cognitive–affective symptom network for young adults. Each node represents a cognitive (blue) or affective (red) item, and edges represent conditional relationships estimated via the Graphical LASSO method. Edge sign indicates whether associations are positive (green edges) or negative (orange), and edge thickness corresponds to the absolute strength of the relationship. (B) Same as (A) but for older adults.

Cognitive–affective networks for young and older adults were constructed for young and older adults. In both age groups, nodes clustered into cognitive and affective domains, with predominantly positive connections within each domain and largely negative connections between them, although some inter-domain connections were positive (Figure 1).

**Figure 2.**
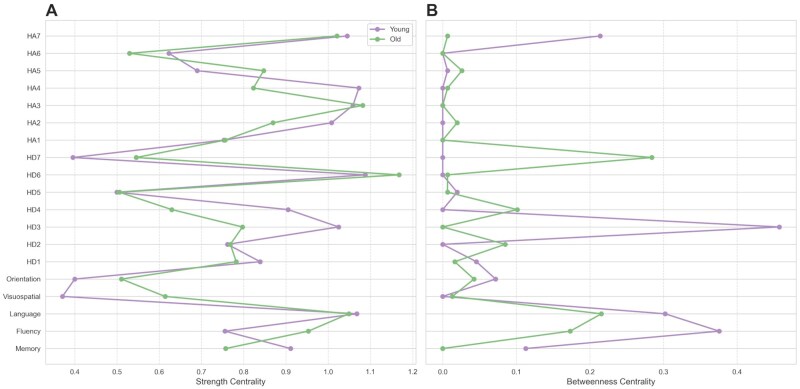
Centrality measures across age groups. (A) Strength centrality for young (purple) and older adults (green). Strength centrality represents the overall connectivity of each node, calculated as the sum of absolute edge weights connected to the node. Higher values indicate nodes with greater influence across the network. The centrality measures were derived from sparsity-based networks, calculated using the Graphical LASSO method with extended Bayesian Information Criteria thresholding. The *x*-axis represents centrality values, and the *y*-axis lists the nodes. (B) Same as (A), but for betweenness centrality for young (purple) and older adults (green). Betweenness centrality represents the importance of a node in a network based on how often it appears on the shortest paths between other nodes. A node with high betweenness centrality acts as a key connector within the network.

To examine the network structure and whether it differs between age groups, we calculated two key centrality measures, namely strength centrality and betweenness centrality. First, strength centrality, which quantifies the overall importance of a node by summing up the overall connection weight of a node, was computed. The pattern of strength centrality values was overall consistent between groups (Figure 2A). The rank of strength centrality values was indeed highly correlated between young and older adults (Spearman’s ρ = 0.795, *p = *4.80e-05). In terms of their values, the strongest cognitive nodes for both young and older adults were memory (young: 0.848, old: 0.731), fluency (young: 0.694, old: 0.883), and language (young: 1.063, old: 0.991). Among the affective nodes, the nodes with the highest strength centrality for both groups were anticipatory anhedonia/enjoyment (HD6) (young: 1.045, old: 1.155) for depression and worrying thoughts (HA3) (young: 1.031, old: 1.070) for anxiety. A permutation test (*n* = 5,000) comparing mean strength centrality between depression-related nodes (HD1–HD7) and anxiety-related nodes (HA1–HA7) showed no significant differences in either age group (young: mean observed difference = −0.217, *p = *.618; old: mean observed difference = −0.207, *p = *.531). Together, these results suggest that the overall network structure was similar between young and older adults.

Next, we computed two independent and complementary bridging measures. First, we computed each node’s betweenness centrality, which quantifies how often a node appears on the shortest paths between other nodes. In contrast to strength centrality, which indicated stability in overall network structure, the betweenness centrality pattern was different between young and older adults (Figure 2B). The rank of betweenness centrality values was not correlated between young and older adults (Spearman’s ρ = 0.330, *p *= .168). In terms of their value, the symptoms with the highest betweenness value were dysphoria (HD3) in young adults (0.4575) but anhedonia (HD7) in older adults (0.2843). A permutation test (*n* = 5,000) comparing the mean betweenness centrality between depression- and anxiety-related nodes showed group-specific differences. Specifically, in young adults, depression and anxiety nodes did not differ significantly (observed difference = 0.053, *p = *.702). However, in older adults, betweenness centrality was significantly higher for depression-related nodes compared to anxiety-related nodes (observed difference = 0.326, *p = *.033).

The second bridging measure we computed was BEI, which quantifies bridging based on predefined node (symptom) labels. The analysis showed similar results to those of betweenness centrality above (Supplementary Figure 1; see online supplementary material), namely no rank correlation between BEI in young and older adults (Spearman’s ρ = .326, *p *= .173); dysphoria (BEI = −0.0741) and anhedonia (BEI = −0.2012) had the highest negative BEI values in young and older adults, respectively; and no differences in young adults (mean observed difference = −0.071, *p = *.548) but significantly higher depression-related BEI compared to anxiety-related BEI in older adults (mean observed difference = −0.443, *p = *.0014). Interestingly, rumination (HA3) showed a surprisingly positive BEI value in both young (0.0314) and older adults (0.0516).

To assess whether these network patterns reflect a gradual age-related shift, we conducted an additional network analysis in middle-aged adults. The midlife network resembled those of both younger and older groups (Supplementary Section 2, Supplementary Table 2, Supplementary Figures 2–3; see online supplementary material). Importantly, the network in middle-aged adults showed stronger associations between anhedonia and cognitive nodes than in younger adults, similar to the pattern seen in older adults.

To complement our findings from network analyses, we tested the latent structure of affective and cognitive factors using an exploratory factor analyses in the different age groups (Supplementary Section 3, Supplementary Table 3; see online supplementary material). The factor analyses showed a broadly similar separation between cognitive and affective factors in young and older adults, with dysphoria loading primarily on the affective factor and anhedonia showing increasing loading on cognition in older age. In the middle-aged group, anhedonia loaded highly on cognition, but the cognitive factor itself explained less variance than in older adults. Together, the factor analyses are consistent with the symptom network analyses, suggesting an age-related shift from dysphoria to anhedonia in linking affective and cognitive factors.

### Bridging symptoms, GMV, and cognitive performance

**Figure 3.**
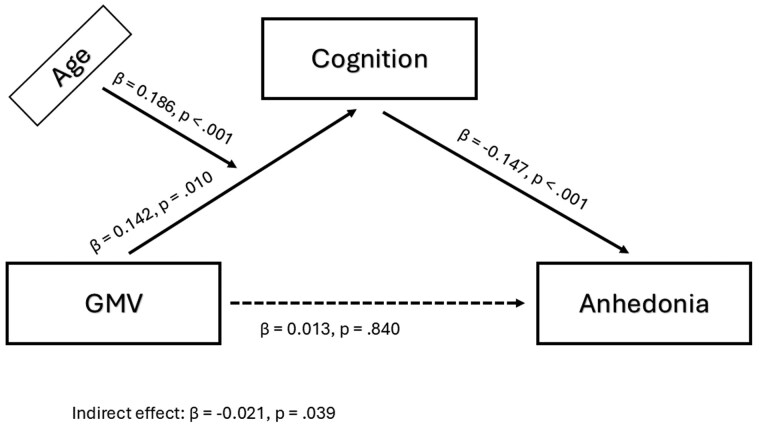
Age-moderated mediation of the GMV–anhedonia association via cognition. Moderated–mediation model in which cognition (ACE-R total) mediates the association between gray-matter volume normalized by total intracranial volume (GMV/TIV; shown as GMV in the diagram) and anhedonia (HD7; shown as Anhedonia). Age moderates the path from GMV to cognition. Boxes represent observed variables; solid lines denote structural paths; the dashed line denotes the direct effect from GMV to anhedonia. Values on paths are standardized coefficients (β) with two-tailed *p*. The indirect effect via cognition is reported below the diagram. All continuous variables were *z*-scored; models adjust for sex and education. ACE-R, Addenbrooke’s Cognitive Examination—Revised.

To examine whether the age-related difference in bridging symptoms moderates the relationship between GMV and cognitive performance, we tested for a moderated mediation using structural equation model. The results of the model are summarized in Table 2. In the model, cognition mediated the association between GMV and depressive symptoms, with age moderating the path from GMV to cognition (Figure 3). The model showed a robust positive association between GMV and cognition, a negative association between cognition and anhedonia (HD7), and no residual direct association between GMV and HD7 once cognition was included. Instead, the association between GMV and HD7 was explained through their association with cognition. The corresponding mediation for dysphoria (HD3) was not supported by the data, and age significantly moderated the path from GMV to cognition. Complementary item-level regression and multivariate moderation analyses confirmed that the observed age-related bridging effect was driven specifically by the coupling between anhedonia and cognition, rather than dysphoria (Supplementary Section 4, Supplementary Table 4; see online supplementary material).

**Table 2.** Moderated–mediation SEM of gray-matter volume, cognition, and depressive symptoms.

Path	β	95% CI	*p*
**Anhedonia (HD7)**			
** GMV → cognition (a)**	0.142	[0.013, 0.264]	.010
** GMV × age → cognition (moderation on a)**	0.186	[0.108, 0.258]	<.001
** Cognition → HD7 (b)**	−0.147	[−0.248, −0.050]	<.001
** GMV → HD7 (c′)**	0.013	[−0.092, 0.119]	.840
** Indirect via cognition (mean age)**	−0.021	[−0.051, −0.001]	.039
** Index of moderated mediation**	−0.027	[−0.048, −0.010]	.003
**Dysphoria (HD3)**			
** GMV → cognition (a)**	0.142	[0.013, 0.264]	.010
** GMV × age → cognition (moderation on a)**	0.186	[0.108, 0.258]	<.001
** Cognition → HD3 (b)**	−0.049	[−0.139, 0.027]	.259
** GMV → HD3 (c′)**	−0.029	[−0.136, 0.077]	.646
** Indirect via cognition (mean age)**	−0.007	[−0.023, 0.005]	.301
** Index of moderated mediation**	−0.009	[−0.026, 0.005]	.268

*Note*. Standardized path estimates for the structural equation model are presented for anhedonia (HD7) and dysphoria (HD3). The model specifies cognition (ACE-R total) as a mediator of the association between gray-matter volume normalized by total intracranial volume (GMV/TIV; reported as “GMV” in the table) and depressive symptoms, with age modeled as a moderator of the path from GMV to cognition. Values are reported as standardized coefficients (β), two-tailed *p* values, and 95% percentile bootstrap confidence intervals (5,000 resamples). Indirect effects are summarized at the sample mean of age, and the index of moderated mediation quantifies the change in the indirect effect per one standard-deviation increase in age. All continuous variables were z-scored; models adjust for sex and education. Model fit: χ^2^(1) = 31.47, *p* <.001; CFI = 0.891; RMSEA = 0.204 [90% CI: 0.146, 0.268]; SRMR = 0.039; *R*^2^(cognition) = 0.285. CFI = comparative fit index; RMSEA = root mean square error of approximation; SRMR = standardized root mean square residual.

SEM, structural equation model; GMV, gray matter volume; ACE-R, Addenbrooke’s Cognitive Examination—Revised; TIV, total intracranial volume.

## Discussion

Our study investigated the structure of the cognitive–affective symptom network across the lifespan. Overall network structure was similar across groups; however, distinct patterns of symptom connectivity emerged for the bridging of affective and cognitive symptoms between age groups. Across two independent bridging measures, dysphoria was the strongest cognitive–affective bridging symptom in young adults, while anhedonia was the strongest bridging symptom for older adults. Moreover, depressive symptoms had significantly higher cognitive–affective bridging values compared to anxiety symptoms, specifically for older adults. Moreover, a larger anhedonia-to-dysphoria difference was associated with a stronger and more positive relationship between GMV and cognition, with this effect being most pronounced in older adults. These findings suggest an age-related shift in the symptoms that bridge cognitive and affective symptoms.

### Model-free symptom severity

Cognitive and anxiety items showed a distinct age-related pattern, with anxiety symptoms being more severe in younger adults, while cognitive function was better preserved in this group compared to older adults.

This pattern reflects the generally higher prevalence of anxiety in younger populations within the general population, consistent with prior epidemiological findings (Kessler et al., 2010; Remes et al., 2016). In contrast, depressive symptoms exhibited greater variability, consistent with the large heterogeneity of depression manifestation (Fried, 2017; Goldberg, 2011). Older adults reported more severe psychomotor slowing, while younger adults reported more severe mood-related symptoms. These findings are consistent with known age-related differences in depressive symptom profiles (Schaakxs et al., 2017; Sutin et al., 2013), and our principal aim was to examine cognitive–affective interactions by age using symptom network analyses.

### Maintenance of the overall cognitive–affective network structure

Strength centrality, which reflects the overall connectivity of nodes, showed remarkable stability across age groups. Within the cognitive nodes, memory and language consistently retained high centrality values in both young and older adults. This likely reflects their role as core cognitive functions that remain relevant regardless of age. Similarly, within the affective nodes, anticipatory anhedonia (HD6) and worrying thoughts (HA3) retained high centrality values across age groups. This pattern aligns with previous findings indicating that the core organization of depressive and anxiety symptom networks, including the centrality of key symptoms, remains largely stable across the lifespan (Harlev et al., 2025b).

This cross-domain consistency across the lifespan supports the view that affect and cognition form an integrated clinical construct that is preserved with age. Indeed, meta-analytic evidence confirms that depressive symptoms are reliably associated with cognitive control deficits from childhood through old age (Dotson et al., 2020). This stability suggests that depression, anxiety, and cognitive symptoms belong to a common clinical syndrome that remains consistent across age. But while the structure appears similar, the mechanisms linking these domains may change with age, pointing to potentially distinct pathways.

### Age-related shift in cognitive–affective bridging symptoms

Using two independent and complementary bridging centrality measures (betweenness and BEI), we identified a difference in how affective symptoms integrate with cognition across age groups. In younger adults, mood-related symptoms, and particularly cheerful feelings, were strongly connected to cognitive nodes, reflecting the importance of emotional positivity in cognitive performance during earlier life stages (Knight et al., 2016). In contrast, in older adults, anhedonia became the principal bridging symptom, reflecting its stronger association with cognitive deficits in late life (Regier et al., 2013).

A shift from a mood-related link to cognitive performance in younger adults to anhedonia-related link to cognitive performance in older adults may reflect age-related neurobiological changes in affective regulation. Whereas younger adults show strong reciprocal links between mood and cognitive function (Elliott et al., 2011), aging is associated with reduced emotional responsivity but increased motivational deficits (Husain & Roiser, 2018). Consistent with this, in Late-Life Depression, apathy—but not dysphoria—exhibits the strongest association with cognitive dysfunction (Harlev et al., 2025a).

Building on these results, we tested a moderated mediation model to examine whether age influences the links between brain structure, cognition, and depressive symptoms. Cognition (ACE-R total) mediated the association between GMV and depressive symptoms, while age moderated the indirect path from GMV to cognition. This pattern indicates that in younger adults, structural variation in GMV has little bearing on cognition or affect, whereas in older adults, lower GMV is increasingly associated with reduced cognitive performance, which in turn predicts greater anhedonia. In other words, with advancing age, structural brain differences are increasingly associated with differences in affective symptoms through cognitive pathways. These results are consistent with observations that in older adults, anhedonia is more closely linked to cognitive decline and structural brain changes (Turner & Husain, 2022), while in young adults, emotional changes are more closely linked to cognition (Scher et al., 2005). Two complementary strands of evidence reinforce this interpretation. A large-scale population study shows that anhedonia is associated with differences across multiple structural brain indices, including total gray and white matter volume, subcortical volumes, cortical thickness, and white matter integrity (Zhu et al., 2021). Clinical data further indicate that variation in brain structure in the context of depression is associated with cognitive performance (Capogna et al., 2019). In line with this evidence, our results indicate that in later life, structural differences relate to depressive symptoms through cognition, most clearly for anhedonia.

Beyond brain structural differences, network-based measures further highlight this shift in cognitive–affective interactions with aging. While overall network strength remained comparable between depressive and anxiety symptoms, depressive symptoms in older adults exhibited stronger connectivity to cognitive nodes compared to anxiety symptoms. This finding underscores the increasingly tight link between depressive symptoms and cognitive functioning in older populations (Jellinger, 2023; Koenig et al., 2014).

Together, these findings suggest that in aging, depression—particularly its anhedonic aspects—may be increasingly intertwined with cognitive decline and underlying brain vulnerability. Indeed, apathy and anhedonia have been increasingly recognized as key early features of neurodegenerative conditions, including Alzheimer’s disease, Parkinson’s disease, and frontotemporal dementia (Steffens et al., 2022). Their presence in these disorders reflects disruptions in reward-processing circuits, which are crucial for both cognitive and affective regulation (Husain & Roiser, 2018). In aging, anhedonia may represent a distinct mechanism of depression—one more closely tied to neurodegenerative pathways than to mood dysregulation.

### Potential clinical implications: rethinking late-life depression treatment

Our findings, derived from a healthy population-based cohort, cannot directly inform treatment guidelines. Nonetheless, they raise the possibility that anhedonia in aging may represent a distinct mechanism of depression, one that is more closely tied to neurodegenerative pathways than to mood dysregulation. Currently, depression in older adults is treated similarly to depression in younger adults, despite lower efficacy (Unützer, 2007). While serotonin-based antidepressants remain standard, anhedonia and apathy may be more strongly linked to dysfunction in dopamine-mediated reward circuits (Alexopoulos, 2019). Dopaminergic interventions, cognitive-behavioral strategies emphasizing goal-directed activity, and neurostimulation approaches have shown promise in targeting motivational impairments rather than mood dysregulation alone (Yuen et al., 2015). Future research should investigate whether such approaches can prove efficacious in older adults suffering from anhedonic depression.

### Strengths and limitations

To our knowledge, few studies have directly compared cognitive and affective symptoms in large population-based samples. By doing so, our study demonstrated both stable and dynamic patterns in the cognitive–affective network. Moreover, by integrating neuroimaging data, the analysis provided supplementary evidence for a possible biological link underlying cognitive–affective interactions with age.

However, several limitations must be considered. First, the cross-sectional design precludes causal inferences. Our findings describe associations between symptoms, cognition, and GMV, and cannot be interpreted as evidence of underlying mechanisms. Second, our neuroimaging analyses focused on global GMV rather than region-specific effects, providing a standardized clinical measure but limiting insights into the role of specific brain structures in cognitive–affective interactions. Third, the study did not include a clinical population, which restricts the generalizability of findings to clinical settings. However, we argue this may better reflect patterns in the general population. Symptom levels were also predominantly low, as expected in a population-based cohort. Nonetheless, the use of LASSO regularization provides robustness to such sparse endorsements. Fourth, reliance on self-reported symptom measures could introduce biases, particularly in older adults, which would affect the accuracy of symptom network measures. This limitation is further highlighted by the interpretation of single-item measures, which may not fully capture the complexity of syndromes like anhedonia and apathy. This oversimplification, while common to many other symptom network studies, calls for caution when interpreting the results of these studies.

### Conclusion

Depressive symptoms, and particularly anhedonia, become more central in linking cognitive and affective symptoms with age. Higher anhedonia relative dysphoria was associated with a stronger and more positive relationship between GMV and cognition, particularly in older adults. These findings highlight how aging alters cognitive–affective interactions and underscore the need for therapeutic strategies that account for both symptom profiles and neurobiological factors across the lifespan.

## Supplementary Material

gbaf252_Supplementary_Data

## Data Availability

All data used for this work are publicly available upon signing data sharing agreement on https://cam-can.mrc-cbu.cam.ac.uk/dataset/. Code used to analyze the data and generate the figures is available on https://github.com/dharlev/From-dysphoria-to-anhedonia-Age-related-shift-in-the-link-between-cognitive-and-affective-symptoms.
