# Supplementary material for: From dysphoria to anhedonia: age-related shift in the link between cognitive and affective symptoms
Source: J Gerontol B Psychol Sci Soc Sci. 2025 Dec 10;81(2):gbaf252. doi: 10.1093/geronb/gbaf252 (PMC12815602; doi:10.1093/geronb/gbaf252)
Supplement: gbaf252_Supplementary_Data [file gbaf252_supplementary_data.zip › JGPS suppl Harlev, Vituri, Shahar, & Wolpe.docx]

***The Journals of Gerontology, Series B: Psychological Sciences and Social Sciences* Supplementary Material: Harlev, Vituri, Shahar, & Wolpe. From dysphoria to anhedonia: Age-related shift in the link between cognitive and affective symptoms.**

**Supplementary Analyses**

*1. Symptom distributions, severity strata, and zero inflation*

Symptom distributions for HADS depression and anxiety totals are summarized separately for each age group (Supplementary Table 1). In the full sample (n = 2598), depression scores had a mean of 3.31 (SD = 2.90, range 0–17), with 91.1% of participants classified in the *“none”* range according to standard HADS cutoffs (0–7), 6.1% as mild (8–10), 2.4% as moderate (11–14), and 0.4% as severe (15–21). Anxiety scores had a mean of 5.16 (SD = 3.39, range 0–20), with 78.6% in the *“none”* range (0–7), 13.8% mild, 6.2% moderate, and 1.4% severe.

Age comparisons showed that young adults reported lower depression (M = 2.92, SD = 2.80) and higher anxiety (M = 5.92, SD = 3.43) compared to older adults (M depression = 3.70, SD = 2.83; M anxiety = 4.50, SD = 3.22), with middle aged participants showing intermediate values (M depression = 3.01, SD = 3.06; M anxiety = 5.53, SD = 3.43).

We used zero-inflation tests to test whether the number of observed zeros was higher than what would be expected at random (Poisson distribution). In the full sample, depression total scores showed 12.8% zeros observed vs. 3.7% expected (Z = 24.90, p < 0.001), and anxiety total scores showed 5.2% observed vs. 0.6% expected (Z = 30.89, p < 0.001). Similar patterns were present within each cohort (Supplementary Table 1). These findings demonstrate both the low symptom levels typical of population-based samples and the presence of excess zeros relative to distributional expectations. Importantly, the analytic approach adopted in the main text (LASSO regularization) is well-suited to such data, as it is robust to sparse symptom endorsements.

*2. Middle-aged symptom–cognition network: structure, centrality, and bridging*

We estimated a symptom–cognition network for the middle aged group (46–64 years, n = 545), alongside the young (≤45 years; n = 756) and older (≥65 years; n = 1230) participants, using the same preprocessing and analytical approach as in the main analyses, based on Gaussian graphical models estimated via graphical LASSO, yielding sparse networks of conditional associations between symptoms and cognition (edges reflecting regression coefficients), with model selection by EBIC (Extended Bayesian Information Criterion) and centrality metrics computed as in the main analyses. As shown in Supplementary Table 2, symptom distributions in the middle-aged group were overall closer to those of the young cohort than to those of the older cohort. Nevertheless, the cognitive-affective network in middle-aged adults showed a well-defined separation between cognitive and affective items, resembling the overall organization observed in both younger and older groups (Supplementary Figure 2). Moreover, in both betweenness and bridge expected influence (BEI), HD1 and HD7 (anhedonia) emerged as the most prominent bridging symptoms, closely resembling the older profile and diverging from the young (Supplementary Figure 3). Strength centrality ranks confirmed overall similarity of network structure, with significant Spearman correlations of ρ = 0.79 (p = 4.8e–05) for young–older, ρ = 0.76 (p = 1.4× e–05) for young–middle age, and ρ = 0.68 (p = 0.0014) for middle age–older. By contrast, betweenness ranks showed no concordance across groups (all ρ < 0.20, all p > 0.42). Taken together, middle-aged participants resembled young adults in raw symptom levels, but their bridging symptoms were more similar to the pattern observed in older adults.

*3. Exploratory factor analyses across age groups*

Exploratory factor analyses were conducted separately for the young, middle-aged, and older groups on 19 variables (seven depression items, seven anxiety items, and five cognition indices). Each analysis used oblique (oblimin) rotation to allow for correlated factors. The number of factors to retain was determined by parallel analysis based on 5,000 random datasets matched for sample size and dimensionality. In each group, the observed eigenvalues of the first three components exceeded those from the simulated datasets, whereas the remaining components did not, indicating that only three factors captured meaningful shared variance among the variables. Thus, a three-factor solution was favoured for all age groups, with the first three eigenvalues as follows: young 4.44, 2.02, and 1.44; middle-aged 4.91, 1.98, and 1.35; older 4.12, 2.52, and 1.55. Factor loadings for the three factors are reported for each group in Supplementary Table 3. In all three age groups, the first factor was affective. In the young group, the cognitive factor explained less of the overall variance, and showed low loadings from non-cognitive items, mainly from dysphoria (HD3). This suggests low cognitive-affect links, with relatively higher links for dysphoria, as found in the network analysis. In the middle-aged group, variance in the affective items still accounted for most of the overall variance, but the pattern of loadings changed: anhedonia (HD7), rather than dysphoria (HD3), showed some loading on the cognitive factor, which was smaller than the loading of HD7 on the cognitive factor in the older adult group, but higher than its loading on cognition in the young adult group. In the older group, the cognitive factor explained more variance, with anhedonia loading strongly on this factor. Taken together, these complementary results support the findings from the network analyses. Specifically, in young adults, cognition is only weakly related to affective symptoms and primarily to dysphoria, whereas in middle-aged adults, the association between cognition and affective symptoms is mainly through anhedonia, as seen in older adults.

*4. Targeted follow-up tests clarifying the bridged relation*

To further specify the link reflected in bridging, we estimated item-level models in which cognition was regressed on each affective item, age, and their interaction, controlling for sex and education. Each model took the form:

ACEᵢ = β₀ + β₁(Itemᵢ) + β₂(Ageᵢ) + β₃(Itemᵢ×Ageᵢ) + β₄(Sexᵢ) + β₅(Educationᵢ) + εᵢ

The results are summarised in Supplementary Table 4. Item-level models confirmed that the age-related increase in the link between cognition and affect (interaction term) was specific to anhedonia (HD7), with smaller effects for HD4 and HD2, and minimal effect for dysphoria (HD3).

**Supplementary Tables**

**Supplementary Table 1. Affective symptom distributions, severity strata, and zero-inflation across age groups.**

| Cohort | Dep  mean (SD) | Anx  mean (SD) | Dep %  None/Mild/Moderate/Severe | Anx %  None/Mild/Moderate/Severe | Dep zeros, statistic, *p-*value | Anx zeros, statistic, *p-*value |
| --- | --- | --- | --- | --- | --- | --- |
| Full sample (N=2,598) | 3.31 (2.90) | 5.16 (3.39) | 91.1/6.1/2.4/0.4 | 78.6/13.8/6.2/1.4 | 12.8%  (Z=24.90, p<0.001) | 5.2%  (Z=30.89, p<0.001) |
| Young  (≤45, n=756) | 2.92 (2.80) | 5.92 (3.43) | 92.3/5.7/1.7/0.3 | 71.2/18.3/9.0/1.6 | 18.1%  (Z=15.47, p<0.001) | 2.6%  (Z=12.66, p<0.001) |
| Middle age  (46–64, n=612) | 3.01 (3.06) | 5.53 (3.43) | 91.7/4.9/2.5/1.0 | 76.8/14.5/6.2/2.5 | 17.8%  (Z=14.76, p<0.001) | 2.6%  (Z=8.72, p<0.001) |
| Older  (≥65, n=1,230) | 3.70 (2.83) | 4.50 (3.22) | 90.0/7.0/2.8/0.2 | 84.1/10.7/4.5/0.7 | 7.1%  (Z=10.37, p<0.001) | 8.0%  (Z=23.00, p<0.001) |

Note. Zero-inflation tested by comparing observed vs. Poisson-expected zero proportions (Z tests) in depressive (Dep) and anxiety (Anx) items. Severity strata based on HADS cutoffs (None = 0–7; Mild = 8–10; Moderate = 11–14; Severe = 15–21).

**Supplementary Table 2. Demographic, affective symptom, and cognition in young, middle-aged, and older adult groups.**

| Variable | Young (n=756) | Middle age (n=695) | Old (n=1230) | Test Stat (M vs. Y) | FDR-*p* (M vs. Y) | Test Stat (M vs. O) | FDR-*p*  (M vs. O) |
| --- | --- | --- | --- | --- | --- | --- | --- |
| HD1 | 0.46 ± 0.67 | 0.42 ± 0.69 | 0.61 ± 0.78 | 2221570 | 0.22 | 3223140 | 2.41e-08 |
| HD2 | 0.26 ± 0.50 | 0.28 ± 0.53 | 0.27 ± 0.54 | 2346260 | 0.60 | 3826510 | 4.49e-01 |
| HD3 | 0.35 ± 0.54 | 0.31 ± 0.57 | 0.27 ± 0.52 | 2209090 | 0.19 | 3877490 | 1.98e-01 |
| HD4 | 0.70 ± 0.72 | 0.90 ± 0.80 | 1.36 ± 0.93 | 2615130 | 0.00 | 273451.50 | 6.15e-25 |
| HD5 | 0.50 ± 0.74 | 0.46 ± 0.74 | 0.47 ± 0.71 | 2249170 | 0.41 | 3712070 | 5.67e-01 |
| HD6 | 0.37 ± 0.65 | 0.42 ± 0.70 | 0.53 ± 0.72 | 238185.50 | 0.37 | 3426270 | 3.93e-04 |
| HD7 | 0.28 ± 0.63 | 0.21 ± 0.56 | 0.19 ± 0.52 | 2209540 | 0.12 | 3821300 | 4.26e-01 |
| HA1 | 1.06 ± 0.65 | 1.03 ± 0.60 | 0.78 ± 0.62 | 226860.50 | 0.52 | 4524920 | 2.06e-17 |
| HA2 | 0.69 ± 0.81 | 0.61 ± 0.79 | 0.53 ± 0.74 | 2200780 | 0.03 | 3978630 | — |
| HA3 | 0.98 ± 0.87 | 0.91 ± 0.84 | 0.76 ± 0.80 | 221209.50 | 0.22 | 414685.50 | 2.08e04 |
| HA4 | 0.84 ± 0.68 | 0.73 ± 0.68 | 0.60 ± 0.63 | 2110650 | 0.01 | 4124580 | 2.99e-04 |
| HA5 | 0.61 ± 0.64 | 0.56 ± 0.63 | 0.43 ± 0.59 | 221221.50 | 0.22 | 4194030 | 7.58e-06 |
| HA6 | 1.21 ± 0.90 | 1.12 ± 0.85 | 0.92 ± 0.82 | 2209560 | 0.22 | 4274960 | 9.32e-07 |
| HA7 | 0.54 ± 0.67 | 0.56 ± 0.72 | 0.49 ± 0.65 | 231942.50 | 0.93 | 3918450 | 1.33e-01 |
| Memory | 0.93 ± 0.10 | 0.92 ± 0.10 | 0.82 ± 0.17 | 218776.50 | 0.19 | 5268570 | 3.10e-44 |
| Fluency | 0.89 ± 0.13 | 0.90 ± 0.13 | 0.78 ± 0.19 | 235531.50 | 0.60 | 5272060 | 1.15e-44 |
| Language | 0.94 ± 0.10 | 0.96 ± 0.08 | 0.92 ± 0.10 | 254810.50 | 0.00 | 4881330 | 2.47e-26 |
| Visuospatial | 0.98 ± 0.05 | 0.97 ± 0.06 | 0.91 ± 0.14 | 214501.50 | 0.01 | 4888940 | 5.79e-29 |
| Orientation | 0.97 ± 0.06 | 0.96 ± 0.07 | 0.93 ± 0.10 | 2324640 | 0.90 | 472141.50 | 1.86e-21 |
| Sex (%female / %male) | 57.1 / 42.9 | 54.2 / 45.8 | 56.5 / 43.5 | 13 | 0.41 | 0.75 | 4.26e-01 |
| Education (%0/%1/%2/%3) | 1.7 / 4.1 / 8.1 / 86.1 | 7.4 / 5.4 / 5.9 / 81.4 | 29.5 / 7.7 / 3.1 / 59.7 | 29.71 | 0.00 | 130.12 | 2.68e-27 |

Note. Values for each variable represent mean ± SD unless stated otherwise. Statistic for continuous variables is Z from for Middle age vs. Young (M vs. Y) and for Middle age vs. Old (M vs. O); Statistic for categorical variables is from χ² tests. All *p-*values were FDR-corrected.

**Supplementary Table 3. Exploratory factor analysis across age groups.**

| Cohort | Total variance explained (%) | Cognition factor (rank, % variance) | Depressive item cross-loading (loading) | Range of other depressive cross-loadings on cognition factor |
| --- | --- | --- | --- | --- |
| Young (≤45, n=756) | 41.6 | 3rd (10.9%) | HD3 (0.25) | 0.05–0.12 |
| Middle age  (46–64, n=545) | 43.3 | 3rd (10.9%) | HD7 (0.19) | 0.06–0.14 |
| Older (≥65, n=1,230) | 43.1 | 2nd (13.7%) | HD7 (0.38) | 0.01–0.21 |

Note. Principal-axis factoring with varimax rotation; factor retention guided by parallel analysis with 200 random datasets matched on *N* and *p*. Three-factor solutions were retained in all cohorts. Table reports the total variance explained across the three retained factors, the rank position of the cognition factor and the percentage of variance it explained, the strongest depressive cross-loading on this factor (with loading value), and the loading range of the other depressive symptoms on this factor.

**Supplementary Table 4. Item-level regressions predicting cognition with age interactions (HD1–HD7, HA1–HA7).**

| Item | β(Item) | β(Age) | β(Item×Age) | *p*(Item) | *p*(Age) | *p*(Item×Age) | FDR *p*(Item×Age) | Adj. R² |
| --- | --- | --- | --- | --- | --- | --- | --- | --- |
| HD1 | -0.12 | -0.19 | -0.04 | 3.96e-17 | 8.20e-43 | 0.01 | 0.05 | 0.28 |
| HD2 | -0.08 | -0.20 | -0.04 | 7.80e-08 | 4.74e-47 | 0.01 | 0.04 | 0.26 |
| HD3 | -0.07 | -0.21 | 0.01 | 4.81e-06 | 1.05e-48 | 0.63 | 0.73 | 0.26 |
| HD4 | -0.07 | -0.18 | -0.05 | 2.45e-06 | 5.65e-32 | <0.001 | 0.01 | 0.26 |
| HD5 | -0.04 | -0.20 | -0.01 | 1.09e-03 | 2.72e-46 | 0.29 | 0.41 | 0.25 |
| HD6 | -0.07 | -0.19 | -0.02 | 2.20e-06 | 4.93e-42 | 0.23 | 0.40 | 0.26 |
| HD7 | -0.12 | -0.21 | -0.07 | 4.79e-10 | 4.50e-49 | <0.001 | <0.001 | 0.28 |
| HA1 | -0.02 | -0.21 | 0.01 | 2.62e-01 | 4.33e-46 | 0.46 | 0.58 | 0.25 |
| HA2 | -0.06 | -0.21 | 0.02 | 6.99e-05 | 4.15e-50 | 0.16 | 0.32 | 0.25 |
| HA3 | -0.02 | -0.21 | 0.03 | 1.45e-01 | 1.74e-47 | 0.06 | 0.17 | 0.25 |
| HA4 | -0.04 | -0.21 | 0.00 | 8.03e-03 | 1.35e-47 | 0.78 | 0.84 | 0.25 |
| HA5 | 0.00 | -0.20 | 0.03 | 9.43e-01 | 5.53e-46 | 0.12 | 0.29 | 0.25 |
| HA6 | -0.01 | -0.20 | 0.00 | 6.70e-01 | 7.68e-46 | 0.90 | 0.90 | 0.25 |
| HA7 | -0.06 | -0.21 | 0.02 | 8.49e-05 | 4.38e-49 | 0.26 | 0.40 | 0.25 |

Note. Model per item: ACEᵢ = β₀ + β₁(Itemᵢ) + β₂(Ageᵢ) + β₃(Itemᵢ×Ageᵢ) + β₄(Sexᵢ) + β₅(Educationᵢ) + εᵢ. All continuous variables were z-scored. All *p-*values were FDR-corrected.

**Supplementary Figures**


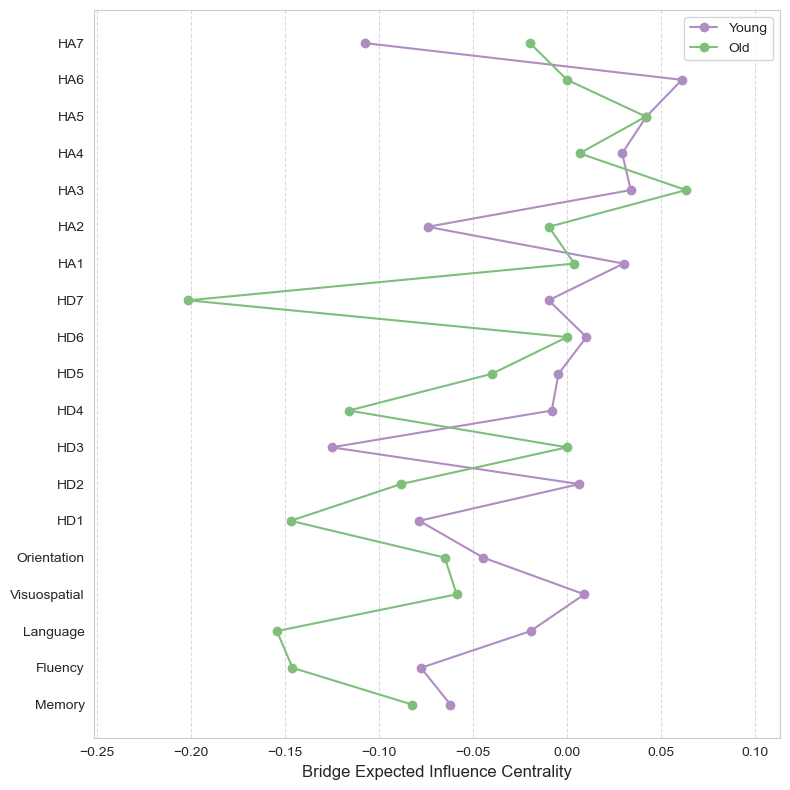


**Supplementary Figure 1. Bridge Expected Influence for all nodes across age groups.** Bridge Expected Influence (BEI) for young (purple) and older adults (green). BEI quantifies a node’s role in explicitly bridging the (labelled) cognitive and affective domains by calculating the sum of edge weights connecting a node to nodes in another domain. Higher values indicate stronger cross-domain connectivity.

**
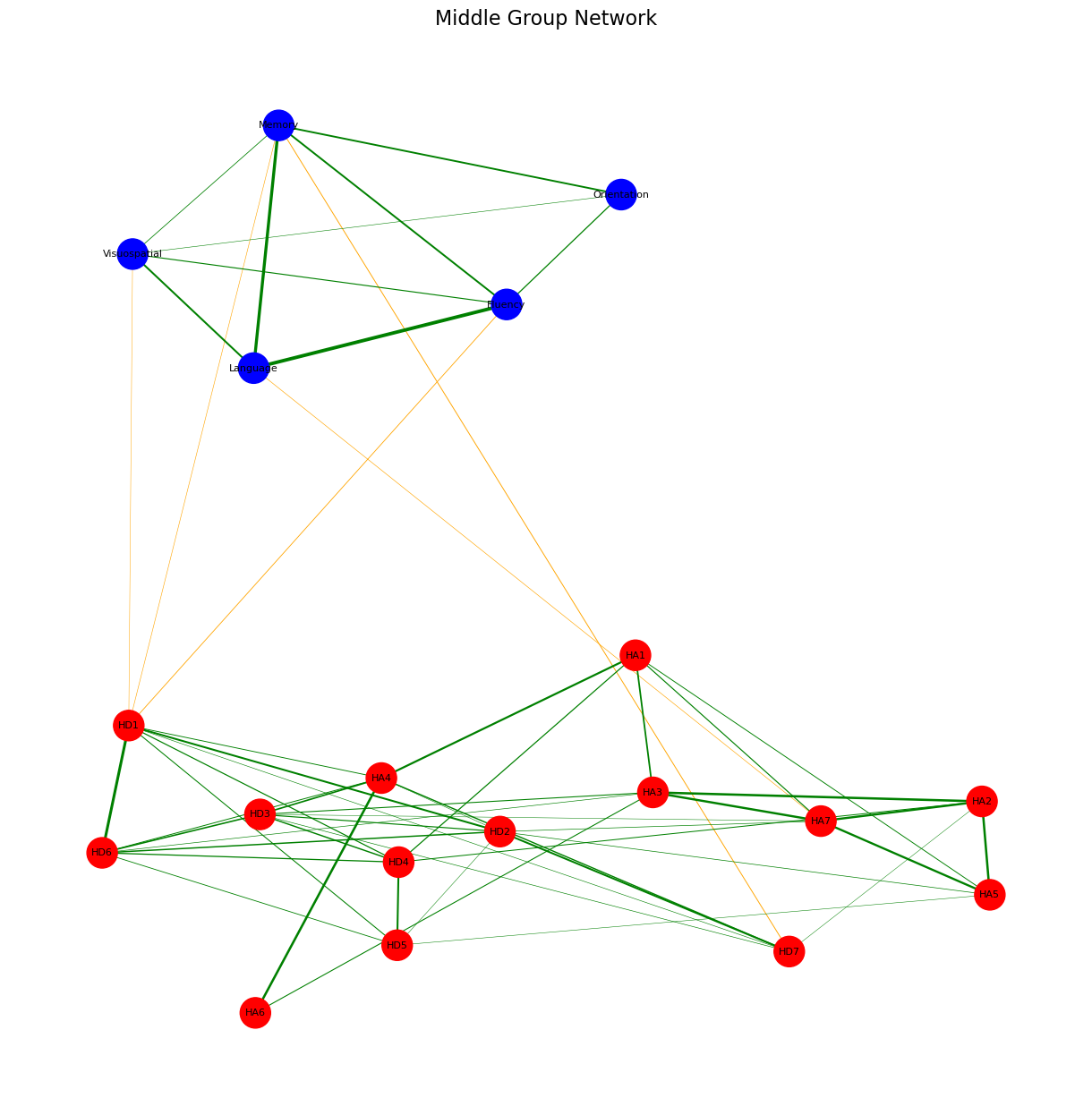
 Supplementary Figure 2. Middle aged group cognitive-affective network.**

Affective symptom–cognition network for the middle-aged group (ages 46–64, n = 545). LASSO-regularized regression network with EBIC (Extended Bayesian Information Criterion) model selection. Nodes in red are Hospital Anxiety and Depression Scale, depression (HD1–HD7) and anxiety (HA1–HA7) items; nodes in blue are ACE-R cognitive indices. Edge thickness indicates absolute weight (regression coefficient). Green edges represent positive coefficients; orange edges represent negative coefficients.

**
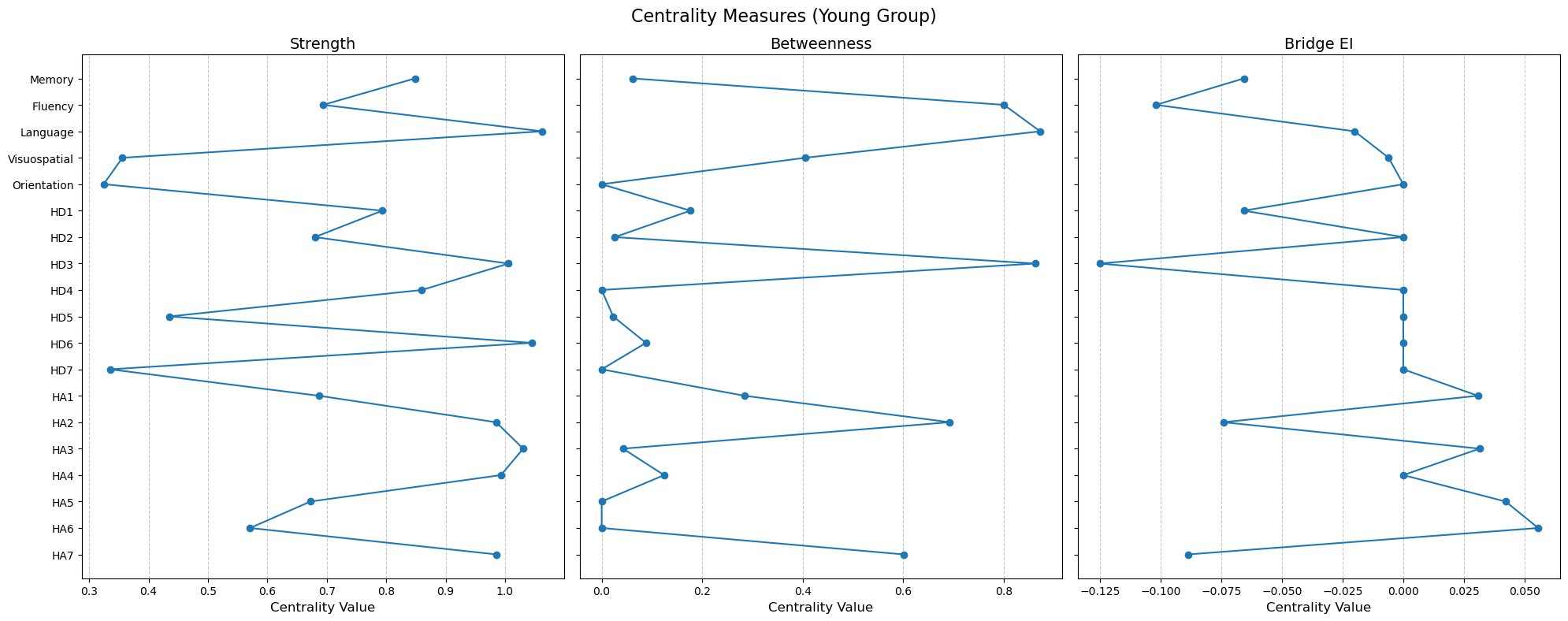
**

**Supplementary Figure 3. Centrality measures for the middle aged group network.**

Strength (left panel), betweenness (centre), and bridge expected influence (right) centrality indices are displayed for each node in the middle aged network.
